# Application of Meridian Electrical Conductance in the Setting of Acute Ischemic Stroke: A Cross-Sectional Study

**DOI:** 10.1155/2019/3098095

**Published:** 2019-08-14

**Authors:** Shih-An Chang, Yi-Xiang Weng, Shu-Chen Cheng, Yeu-Jhy Chang, Tsong-Hai Lee, Chien-Hung Chang, Ting-Yu Chang, Kuo-Lun Huang, Chi-Hung Liu, Chung-Yuan Hsu

**Affiliations:** ^1^Division of Chinese Acupuncture and Traumatology, Center of Traditional Chinese Medicine, Taoyuan Chang Gung Memorial Hospital, Taoyuan, Taiwan; ^2^Stroke Center and Department of Neurology, Chang Gung Memorial Hospital, Linkou Medical Center, Chang Gung University College of Medicine, Taoyuan, Taiwan

## Abstract

Acupuncture is widely used for improving poststroke care. Knowing the condition of meridian can help traditional Chinese medicine (TCM) doctors make a tailored choice of acupoints for every patient. The establishment of an objective meridian energy measurement for acute ischemic stroke that can be used for future acupuncture treatment and research is an important area in stroke-assisted therapy. In this study, a total of 102 subjects diagnosed with acute ischemic stroke within 7 days of onset were recruited, and the meridian energy analysis device (MEAD) was used to record the meridian electrical conductance (MEC) values of twelve meridians on unaffected and affected limbs. We found that the MEC value of the twelve meridians on the affected limbs was significantly higher than that on the unaffected limbs (*P*=0.001). Compared with the unaffected limbs, there was a higher value of MEC on the affected limbs of the lung meridian, heart meridian, pericardium meridian, and small intestine meridian, with significant differences (*P* < 0.05, *P* < 0.001, *P* < 0.001, and *P* < 0.05, respectively). Further analysis revealed that the MEC values of both Yin and Yang meridians of the affected limbs were significantly higher than those of the unaffected limbs (*P*=0.001 and *P* < 0.05, respectively). Meanwhile, the mean of the index of sympathovagal balance in patients with acute ischemic stroke (5.49 ± 4.21) was higher than the normal range (1–1.5), indicating autonomic imbalance. The results of this study are consistent with TCM theory as well as clinical observation and pathological mechanisms, suggesting that the measurement of MEC values may be used as a supplementary diagnostic method for acupuncture in patients with acute ischemic stroke.

## 1. Introduction

The global incidence of stroke is 258 per 100,000 people per year, and it is estimated that approximately 16.9 million patients suffer from this disease each year [1]. Stroke is the 3^rd^ leading cause of death, with a 7.2% mortality rate and long-term disability in Taiwan, and it results in an expenditure of 475 million dollars annually on Taiwan hospital health-care resources [2]. Stroke is categorized into ischemic stroke and hemorrhagic stroke. There is a higher incidence of ischemic than hemorrhagic stroke, accounting for approximately 74% of stroke patients in Taiwan [3]. Stroke causes disability sequelae such as hemiplegia, aphasia, and dysphagia. Clinically, in addition to conventional Western medicine treatment, acupuncture is widely used in the adjuvant treatment of subacute stroke and stroke sequelae. The reduced stroke recurrence rate is also observed in ischemic stroke patients with acupuncture treatment form Taiwan National Health Insurance Research Database [4]. However, the application of acupuncture in the acute phase of stroke is relatively rare in clinical practice [5].

In TCM theory, the etiology of stroke is classified into “Blood stasis,” “Wind,” “Phlegm,” “Qi deficiency,” “Fire-heat,” and “Yin deficiency with Yang hyperactivity” [6]. All of these etiologies can cause the change of meridian energy in different degrees, which is the therapeutic target of acupuncture [7]. It is known that acupuncture can dredge the disturbance of Yin and Yang and balance the energy of meridians, which involves the modulation of autonomic imbalance [8, 9]. TCM doctors tend to choose different treatment in accordance with the patient's individuality. It is important to know the condition of the meridians, which may indicate the choice of acupoints [7, 10, 11]. The excess and deficiency of meridians may change the treatment strategy of acupuncture [12]. Therefore, understanding the condition of the meridians in patients with acute ischemic stroke can provide a basis for clinical acupuncture treatment.

The meridian energy analysis device (MEAD), developed by Dr. Yoshio in the 1950s [13], has become the main instrument for meridian diagnosis. The instrument is based on the Ryodoraku theory and the principle of electronic skin measurement [14]. The values of meridian electrical conductance (MEC) reflect the conditions of the related meridians and organs. Previous studies have revealed that there is higher electrical conductance and potential in acupoints than in the surrounding tissue [15–17], and there is greater electrodermal conduction in the meridians than in the nonmeridians [18]. MEAD has been widely used in meridian studies because it is noninvasive and low in cost and has high reproducibility and stability [19–21]. Many researchers have used MEAD to assess the physiological status, evaluate the effectiveness of the treatment, or conduct clinical trials of TCM treatment [12, 22–28]. In addition, MEAD has been used in the diagnosis of many diseases, such as gastroesophageal structural lesions, tennis elbow pain, low back pain, and obesity [21, 29–31].

Yu-Chih et al. found that patients with stroke sequelae have lower meridian electrical conductance (MEC) values compared with those in healthy people [32]. In recent years, there have been few studies on the meridian energy change in the acute phase of ischemic stroke. Since the condition of acute stroke is complicated and critical, the purpose of this study is to use MEAD to measure the meridian energy change in patients suffering from ischemic stroke within 7 days after onset in an attempt to determine the underlying rules to provide a reference for acupuncture treatment.

## 2. Materials and Methods

### 2.1. Study Design

The protocol was approved by the Human Ethics Committee of the study hospital (Chang Gung Medical Foundation Institutional Review Board no. 104-2081B) and was performed from 1 April 2015 to 9 January 2017. Written and oral informed consent was obtained from all participants at the beginning of the study.

### 2.2. Participants

The participants were recruited from the inpatients diagnosed with acute ischemic stroke in the Neurology Department at Chang Gung Memorial Hospital in Linkou, Taiwan, and all met the inclusion and exclusion criteria described below.

#### 2.2.1. Inclusion Criteria

1. Diagnosis of acute ischemic stroke based on the clinical manifestations and CT or MRI
2. Onset of stroke within 7 days
3. No complications involving other major organs (heart, liver, kidney, and lung)
4. First-ever stroke or recurrent stroke with a Rankin scale score ≤1 before the current stroke
5. Willingness to receive MEAD measurements

#### 2.2.2. Exclusion Criteria

1. Complications of sepsis or other infections
2. Consciousness unclear or impaired cognition
3. Pacemaker implanted
4. Pregnant
5. Bilateral limb weakness or normal muscle power in four limbs

### 2.3. Measures

The TCM doctors who participated in this study all obtained TCM medical licenses and received short-term training in the use of the meridian energy analysis device (MEAD, ME-PRO 6.1.1, Medpex Enterprises, Taiwan).

The participants were asked to remove all metal materials and lie flat and rest for at least 10 minutes before the assessment. Starting with MEAD, one metal cylinder with a clip was held in the left hand of the participant, and a pressure sensitive examination rod moistened with 3% saline was applied sequentially to 24 acupoints in the 12 left meridians and the 12 right meridians (Supplementary Figure 1 and Supplementary Table 1). The MEC values of each acupoint were recorded automatically by MEAD. We defined the limbs with hemiparesis as the affected limbs and the healthy limbs with normal muscle power as the unaffected limbs.

The twelve meridians are symmetrically distributed on both the left side and right side of the body, and each meridian was divided into the Yin meridian or Yang meridian on the basis of its distribution. There are six Yin meridians distributed on the inner region of the limbs, chest, and abdomen, while there are six Yang meridians distributed on the outside region of the limbs, head, and trunk. According to the location of the meridians in the upper or lower limbs, these six Yin and Yang meridians are further subdivided into three Yin meridians of the arm (heart, lung, and pericardium), three Yang meridians of the arm (small Intestine, large Intestine, and triple energizer), three Yin meridians of the leg (liver, kidney, and spleen), and three Yang meridians of the leg (bladder, gallbladder, and stomach) (Supplementary Figure 2).

For MEAD analysis on the basis of the manufacturer's protocol, the index of sympathovagal balance is defined as the ratio of the highest average MEC value of the dorsal or ventral side of four limbs to the lowest average MEC value, which fits the subgroups of three Yin meridians of the arm, three Yang meridians of the arm, three Yin meridians of the leg, and three Yang meridians of the leg (a total of 8 subgroups because both left side and right side are included). This index is calculated automatically by the device and is normally between 1.0 and 1.5. If it is not within this range, there may be autonomic imbalance.

### 2.4. Statistical Analysis

The statistical analysis was performed using SPSS software version 20.0 (IBM, Armonk, NY, USA). Demographic data, including sex, age, personal history, and past medical history, were analyzed by descriptive statistics expressed as the mean ± standard deviation or *n* (%). The MEC values of each meridian were expressed as the mean ± standard deviation. A paired sample *t*-test was used to compare the mean MEC values of unaffected limbs and affected limbs. When the *P* value was less than 0.05, the result was considered statistically significant.

## 3. Result

### 3.1. Patient Characteristics

**Figure 1:**
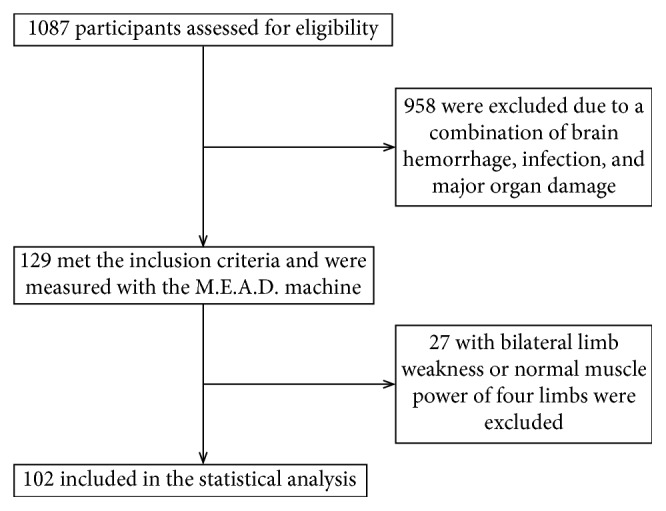
Flow chart of patients included for statistical analysis.

From April 1, 2015, to January 9, 2017, 1087 participants suffered from acute ischemic stroke within 7 days after onset and were assessed for eligibility. After screening, 129 inpatients met the inclusion and exclusion criteria, and their meridian energy changes were measured using MEAD. 27 participants were excluded due to bilateral limb weakness or normal muscle power in four limbs. A total of 102 subjects were included in the statistical analysis, including 59 males and 47 females, with an average age of 65 years. The study flow chart is presented in Figure 1. Other characteristics, such as personal history, past medical history, and the condition of affected limbs, are shown in Table 1.

### 3.2. Primary Outcome

The MEC values of twelve meridians of 102 participants were measured by MEAD. The analysis results of the MEC values are listed in Table 2. The index of sympathovagal balance calculated from these values was 5.49 ± 4.21, indicating that patients with acute ischemic stroke were in a state of autonomic imbalance. The MEC values of the four meridians of the affected limbs were significantly higher than those of the unaffected limbs, including lung meridian (33.43 ± 27.96 vs 28.86 ± 23.97), pericardium meridian (32.50 ± 25.42 vs 27.33 ± 22.52), heart meridian (24.79 ± 21.47 vs 18.91 ± 17.15), and small intestine meridian (26.82 ± 25.12 vs 18.91 ± 17.15) and showed significant differences (*P* < 0.05, *P* < 0.001, *P* < 0.001 and *P* < 0.05, respectively) (Table 2).

### 3.3. Secondary Outcome

TCM doctors tend to choose specific acupoints for poststroke treatment on the basis of different acupuncture faction and theory. A study revealed the acupoints of Yang meridians were applied more frequent than those of Yin meridian [33]. Therefore, the condition of Yin and Yang meridians between unaffected and affected limbs was further analyzed. First, we compared the MEC values of the twelve meridians of the affected and unaffected limbs and found that the MEC values of the twelve meridians of the affected limbs were higher than those of the unaffected limbs (*P*=0.001). Second, we divided the twelve meridians into Yin meridians and Yang meridians for analysis, and we observed that the MEC values of Yin or Yang meridians of the affected limbs were significantly higher than those of the unaffected limbs (*P*=0.001 and *P*=0.02, respectively). Third, we subdivided the Yin and Yang meridians into the Yin meridians of the arm, Yang meridians of the arm, Yin meridians of the leg, and Yang meridians of the leg for analysis. We found that the MEC values of Yin or Yang meridians of the affected arm were significantly higher than those of the unaffected arm (*P*=0.001 and *P*=0.01, respectively), but there was no statistically significant difference in the MEC values of Yin or Yang meridians of the affected leg (*P*=0.93, *P*=0.57, respectively) (Table 3).

## 4. Discussion

We found significant differences in the MEC values between the unaffected and affected sides of patients with acute ischemic stroke. Some interference factors, such as age, sex, underlying disease, body fat, weather, and humidity, may affect the MEC values [12, 27, 28, 34–36]. Xie measured the MEC values of twelve meridians of healthy people, and the results showed that the MEC values of males were higher than those of females [37]. Li discovered that the MEC values were statistically higher in the afternoon than in the morning [20]. On the other hand, it was found that every individual's MEC values had a fixed pattern with a relatively high reappearance rate [19], and there was no statistically significant difference in MEC values between the right and left sides in healthy people [37]. Therefore, instead of using the control group for analysis, we compared the MEC values of the unaffected and affected sides of the same patient to avoid these interference factors and to ensure the stability of the experiment.

Yu-Chih et al. investigated the changes in the MEC values of 82 stroke patients with a sequela of hemiplegia in Taiwan from 1993 to 1996 and found that there was no statistically significant difference in the MEC values between the unaffected and affected limbs [32, 38]. However, the inclusion criteria of the participants in these studies were different from ours in that they comprised a variety of different time courses (from 3 days to 13 years) and types (hemorrhagic and ischemic) of stroke. Because the pathophysiological processes of ischemic and hemorrhagic stroke were dissimilar and the disease condition changed over time, the results of these studies were different [39–41]. Therefore, we selected only patients with ischemic stroke in the acute stage (within 7 days of onset) to minimize the bias of patients' characteristics.

Lin et al. showed that the MEC value of the bladder meridian of the affected side was higher than that of the unaffected side and that the index of sympathovagal balance was high in the patient with acute renal colic [42]. After one month of treatment, the MEC value of the bladder meridian of the affected side decreased to a level similar to that of the unaffected side, with an improvement in sympathovagal balance. Another prospective study of acute renal colic also reported that patients with a ureteral calculus had a higher index of sympathovagal balance than that of the control group, which represented autonomic imbalance [43]. In our study, the MEC values of the affected limbs were significantly higher than those of the unaffected limbs (*P*=0.001), while the mean of the index of sympathovagal balance of patients with acute ischemic stroke (5.49 ± 4.21) was higher than the normal range (1–1.5). As a consequence, we proposed that patients with acute ischemic stroke tend to have higher MEC values on the affected side and autonomic imbalance.

There are 27 patients excluded by bilateral limb weakness or normal muscle power of four limbs. One was found to develop brain abscess and 8 were disclosed that no new-onset stroke by the final MRI report. There is only one stroke patient with bilateral limb weakness, and there are 18 stroke patients with normal muscle power. Hence, we also analyzed the MEC values of twelve meridians of these 18 subjects, and there is no significant difference between right and left limbs (Table 4), but the index of sympathovagal balance remained high (4.20 ± 2.20), indicating that, to some degree, patients suffering from acute ischemic stroke have autonomic imbalance regardless of the involvement of limb weakness.

Researchers have observed that patients with acute ischemic stroke had impaired autonomic function, and the mechanism is still not fully understood [44]. It has also been found that the increased sympathetic activity after acute ischemic stroke was associated with poor prognosis [45, 46]. The hyperactivity of the sympathetic nervous system causes sweat gland secretions, which in turn increases skin conductance. Accordingly, MEAD was used to measure the MEC values at acupoints to assess peripheral autonomic function [38]. We also found a high autonomic imbalance rate in this study (101 of 102 patients, 99.02%).

On the basis of meridian and collateral theory, the body forms a system where Qi and blood circulate through the meridians and collaterals connecting to the viscera, extremities, and superficial organs and tissues [47]. Using MEAD measurements at acupoints can reflect excessive or insufficient meridians [48]. Acupuncture is a treatment method that harmonizes Qi and blood and balances excess and deficiency of meridians as well as Yin and Yang. The therapeutic effect of acupuncture is known as the involvement of autonomic regulation [8, 9]. In other words, acupuncture can modulate the imbalance of parasympathetic and sympathetic activities. Our study discovered that patients with acute ischemic stroke have a relatively high index of sympathovagal balance, which could be selected as a parameter for assessing the efficacy of acupuncture in future studies.

TCM emphasizes that the treatment should be based on the syndrome differentiation. According to the eight principles (Yin, Yang, exterior, interior, cold, heat, deficiency, and excess), syndrome differentiation is used to analyze and distinguish pathological conditions, which is an important component of TCM diagnostics. The vital Qi of the body and the virulence of the pathogenic factors can be determined using the syndrome differentiation of deficiency and excess. In general, the deficiency syndrome refers to the syndrome with deficiency of vital Qi, and the excess syndrome is manifested by exuberant pathogens [49]. Under the principle of diagnosis, most patients with acute stroke represent excess syndrome, while patients with chronic stroke represent mainly deficiency syndrome. A previous study enrolled 253 acute ischemic stroke inpatients (onset within 48 to 72 hours) to analyze TCM syndromes and found that wind, phlegm, and blood stagnation syndromes were the major pathological mechanisms underlying acute ischemic stroke [50]. The wind, phlegm, and blood stagnation syndromes were all classified as excess syndrome. Our study discovered that patients with acute ischemic stroke had higher MEC values in their affected limbs, indicating excessive conditions of the affected limbs. This finding was also consistent with the pathological condition of TCM syndromes.

It is well known that Qi and blood are closely related in TCM theory: “Qi is the commander of blood, and blood is the mother of Qi.” This means that Qi is the motive force of blood, while blood carries Qi and provides nutrients for its movement. The concept of “heart” in TCM controls blood circulation, and “lung” dominates Qi as well as governs Qi diffusion [47]. Tsai discovered that the most common syndrome in acute ischemic stroke was blood stasis (98.4%) [50], indicating that the blood circulation of patients with acute ischemic stroke was blocked. Our study found significant imbalances between the affected and unaffected limbs, particularly in the lung, heart, and pericardium meridians (*P* < 0.05, *P* < 0.001, and *P* < 0.001, respectively). This result provides evidence of close linkages between TCM theory, clinical observation, and pathological mechanisms underlying acute ischemic stroke.

According to our clinical practices, most stroke patients who received acupuncture therapy in the chronic stage had sequela of either flaccid or spastic paralysis. On the basis of TCM theory, because of “treating flaccid paralysis by Yang Ming alone” [51] and “the liver governs the body's sinews,” there is a tendency to choose acupoints of the stomach or liver meridian at first [47]. A study revealed the acupoints of Yang meridians were the major components of the acupuncture prescription for stroke sequelae management (74.7%), with more frequent choice of acupoints in lower limbs (28.1%) than that in upper limbs (22.1%) [33], but our study found the MEC values of both Yin and Yang meridians of the affected arm to be significantly higher than those of the unaffected arm. There was no statistically significant difference in the MEC values of Yin or Yang meridians of the affected leg.

Compared with Huang's research [32, 38], we found that the MEC values of patients with stroke in the acute and chronic stages were different, suggesting that the energy status of meridians in patients with stroke changed with the disease course. Therefore, we believe that acupuncture therapy for stroke patients should be adjusted based on the stage of stroke, and the MEC values may provide us with a reference for the meridian status. In addition, the MEC values of acupoints of the lung, heart, and pericardium meridians may be considered first for acute ischemic stroke. Moreover, due to the significant excess of the affected side meridians, particularly the Yin meridian, an additional strategy using acupuncture to balance the affected and unaffected limbs should also be evaluated.

## 5. Conclusion

We performed MEAD analyses of 102 patients with acute ischemic stroke and found that the MEC values of the affected limbs were higher than those of the unaffected limbs, especially in the lung, heart, and pericardium meridians. In addition, the MEC values of both Yin and Yang meridians of the affected arm were significantly higher than those of the unaffected arm. These patients also had a relatively high index of sympathovagal balance. The results reveal the linkages between the TCM theory, clinical observation, and pathological mechanism underlying acute ischemic stroke and provide a clinical strategy for acupuncture therapy of acute ischemic stroke.

## Figures and Tables

**Table 1:** Participant characteristics.

Characteristic	Value
Age (*y*)	65.38 ± 12.62
Sex	
Male, *n* (%)	59 (55.9)
Female, *n* (%)	47 (44.1)
Personal history	
Smoke, *n* (%)	36 (35.3%)
Alcohol, *n* (%)	20 (19.6%)
Medical history	
Hypertension, *n* (%)	72 (70.6)
Diabetes mellitus, *n* (%)	42 (41.2)
Hyperlipidemia, *n* (%)	36 (35.3)
Arrhythmia, *n* (%)	17 (16.7)
Affected limbs	
Upper limbs, *n* (%)	14 (13.7%)
Lower limbs, *n* (%)	6 (5.9%)
Both upper and lower limbs, *n* (%)	82 (80.4%)

**Table 2:** Comparison of unaffected and affected limbs of each meridian electrical conductance values.

Meridian	Unaffected limbs (*μ*A, mean ± SD)	Affected limbs (*μ*A, mean ± SD)	*P* value
Lung	28.86 ± 23.97	33.43 ± 27.96	0.009^*∗*^
Pericardium	27.33 ± 22.52	32.50 ± 25.42	<0.001^*∗∗*^
Heart	18.91 ± 17.15	24.79 ± 21.47	<0.001^*∗∗*^
Small intestine	22.94 ± 23.82	26.82 ± 25.12	0.03^*∗*^
Triple energizer	25.24 ± 23.09	28.54 ± 26.67	0.09
Large intestine	26.36 ± 24.63	30.11 ± 26.28	0.67
Spleen	19.90 ± 20.85	20.77 ± 21.32	0.24
Liver	17.06 ± 18.58	16.13 ± 18.83	0.33
Kidney	16.16 ± 19.66	16.03 ± 17.98	0.93
Bladder	16.66 ± 17.53	18.27 ± 19.51	0.15
Gall bladder	11.74 ± 13.69	11.18 ± 13.74	0.56
Stomach	15.64 ± 16.84	15.89 ± 18.08	0.83

^*∗*^ *P* < 0.05; ^*∗∗*^ *P* < 0.001.

**Table 3:** Comparison of unaffected and affected limbs of classified meridian electrical conductance values.

Meridian	Unaffected side (*μ*A, mean ± SD)	Affected side (*μ*A, mean ± SD)	*P* value
Twelve meridians	20.57 ± 15.35	22.87 ± 17.57	<0.001^*∗∗*^
Yin meridians	21.37 ± 16.28	23.94 ± 18.59	<0.001^*∗∗*^
Yang meridians	19.77 ± 15.53	21.80 ± 17.56	0.02^*∗*^
Yin meridians of arm	25.03 ± 19.86	30.24 ± 23.55	<0.001^*∗∗*^
Yang meridians of arm	24.85 ± 20.74	28.49 ± 23.89	0.01^*∗*^
Yin meridians of leg	17.71 ± 16.31	17.64 ± 16.58	0.93
Yang meridians of leg	14.68 ± 13.68	15.11 ± 14.36	0.57

^*∗*^ *P* < 0.05 and ^*∗∗*^ *P* < 0.001.

**Table 4:** Comparison of left and right limbs of each meridian electrical conductance in patients with normal muscle power.

Meridian	Left limbs	Right limbs	*P* value
Lung	22.36 ± 24.07	22.63 ± 27.25	0.93
Pericardium	21.96 ± 20.77	22.90 ± 24.41	0.61
Heart	15.16 ± 15.93	16.29 ± 20.58	0.55
Small intestine	21.19 ± 27.48	17.66 ± 19.92	0.39
Triple energizer	20.15 ± 26.96	19.83 ± 20.38	0.94
Large intestine	20.21 ± 24.13	19.50 ± 22.99	0.72
Spleen	20.92 ± 22.88	23.85 ± 24.37	0.06
Liver	12.95 ± 15.17	13.18 ± 14.47	0.95
Kidney	11.13 ± 13.90	20.45 ± 26.17	0.11
Bladder	17.28 ± 23.30	16.66 ± 21.76	0.80
Gall bladder	8.52 ± 11.44	8.29 ± 10.83	0.89
Stomach	13.74 ± 18.61	14.43 ± 13.93	0.88

## Data Availability

The data used to support the findings of this study are included within the article.

## Abbreviations

MEAD: Meridian energy analysis device
MEC: Meridian electrical conductance
TCM: Traditional Chinese medicine.

## Supplementary Materials

Supplementary Materials Supplementary Figure 1: the acupoints used in the measurement of MEC values shown are the locations of acupoints that are used in the measurement of MEC values, including LU-9 = Taiyuan (lung); PC-7 = Daling (pericardium); HT-7 = Shenmen (heart); SI-5 = Yanggu (small intestine); SJ-4 = Yangchi (triple energizer); LI-5 = Yangxi (Large Intestine); SP-3 = Taibai (spleen); LR-3 = Taichong (liver); KI-4 = Dazhong (kidney); BL-65 = Shugu (bladder); GB-40 = Qiuxu (gallbladder); and ST-42 = Chongyang (stomach). Supplementary Figure 2: the distribution of Yin and Yang meridians of the arm and leg. (a) The Yin meridians of the arm are distributed on the inner region of the upper limb (heart, lung, and pericardium). (b) The Yang meridians of the arm are distributed on the outside region of the upper limb (small intestine, large intestine, and triple energizer). (c) Yin meridians of the leg are located in the inner region of the lower limb (liver, kidney, and spleen), and Yang meridians of the leg are located in the outer region of the lower limb (bladder, gallbladder, and stomach). The short dash line represents the yin meridians, and the continuous line represents the yang meridians. Supplementary Table 1: the acupoints used in the measurement of MEC Values.
