# Supplementary material for: Application of Meridian Electrical Conductance in the Setting of Acute Ischemic Stroke: A Cross-Sectional Study
Source: Evid Based Complement Alternat Med. 2019 Aug 14;2019:3098095. doi: 10.1155/2019/3098095 (PMC6710765; doi:10.1155/2019/3098095)
Supplement: Supplementary Materials — Supplementary Figure 1: the acupoints used in the measurement of MEC values shown are the locations of acupoints that are used in the measurement of MEC values, including LU-9 = Taiyuan (lung); PC-7 = Daling (pericardium); HT-7 = Shenmen (heart); SI-5 = Yanggu (small intestine); SJ-4 = Yangchi (triple energizer); LI-5 = Yangxi (Large Intestine); SP-3 = Taibai (spleen); LR-3 = Taichong (liver); KI-4 = Dazhong (kidney); BL-65 = Shugu (bladder); GB-40 = Qiuxu (gallbladder); and ST-42 = Chongyang (stomach). Supplementary Figure 2: the distribution of Yin and Yang meridians of the arm and leg. (a) The Yin meridians of the arm are distributed on the inner region of the upper limb (heart, lung, and pericardium). (b) The Yang meridians of the arm are distributed on the outside region of the upper limb (small intestine, large intestine, and triple energizer). (c) Yin meridians of the leg are located in the inner region of the lower limb (liver, kidney, and spleen), and Yang meridians of the leg are located in the outer region of the lower limb (bladder, gallbladder, and stomach). The short dash line represents the yin meridians, and the continuous line represents the yang meridians. Supplementary Table 1: the acupoints used in the measurement of MEC Values. [file 3098095.f1.docx]

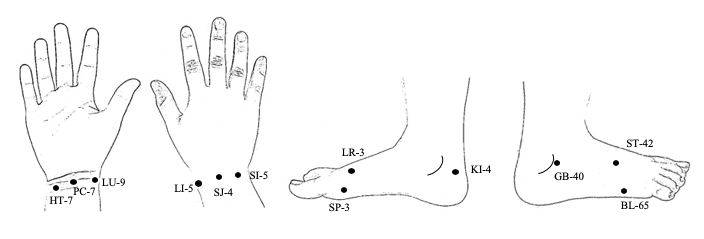


**SUPPLEMENTARY FIGURE 1: The Acupoints Used in the Measurement of MEC Values**

Shown are the locations of acupoints that are used in the measurement of MEC values, including LU-9= Taiyuan(Lung); PC-7= Daling (Pericardium); HT-7= Shenmen(Heart); SI-5= Yanggu(Small Intestine); SJ-4= Yangchi (Triple Energizer); LI-5=Yangxi (Large Intestine); SP-3= Taibai (Spleen); LR-3= Taichong(Liver); KI-4=Dazhong(Kidney); BL-65=Shugu( Bladder); GB-40= Qiuxu (Gallbladder) and ST-42= Chongyang (Stomach).

**
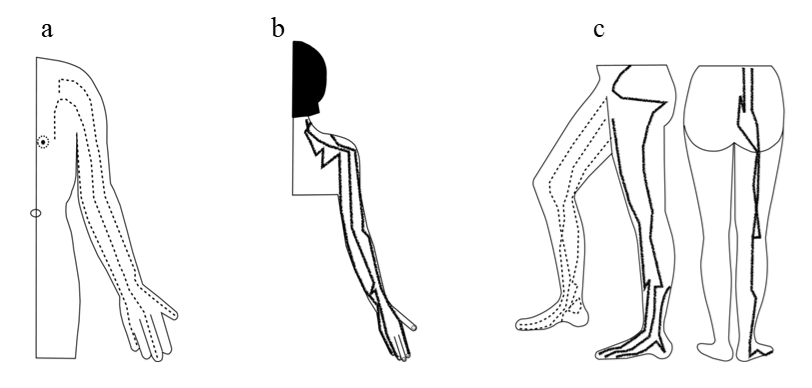
**

**SUPPLEMENTARY FIGURE 2: The Distribution of Yin and Yang Meridians of the Arm and Leg.**

(a) The Yin meridians of the arm are distributed on the inner region of the upper limb (Heart, Lung, and Pericardium). (b) The Yang meridians of the arm are distributed on the outside region of the upper limb (Small Intestine, Large Intestine, and Triple Energizer). (c) Yin meridians of the leg are located in the inner region of the lower limb (Liver, Kidney, and Spleen) and Yang meridians of the leg are located in the outer region of the lower limb (Bladder, Gallbladder, and Stomach). The short dash line represents the yin meridians, and the continuous line represents the yang meridians.

**SUPPLEMENTARY TABLE 1: The Acupoints Used in the Measurement of MEC Values**

| **Acupoints Name** | **Meridian** | **Location** |
| --- | --- | --- |
| LU-9  (Taiyuan) | Lung | On the radial aspect of the palmar wrist crease, in the depression ulnar to the abductor pollicis longus tendon. |
| PC-7  (Daling) | Pericardium | On the anterior aspect of the palmar wrist crease, between the palmaris longus tendon and flexor carpi radialis muscles. |
| HT-7  (Shenmen) | Heart | On the ulnar aspect of the palmar wrist crease, radial to the insertion of the flexor carpi ulnaris tendon. |
| SI-5  (Yanggu) | Small Intestine | On the ulnar aspect of the wrist, in the depression between the triquetrum bone and the ulnar styloid process. |
| SJ-4  (Yangchi) | Triple Energizer | On the dorsum of the wrist , in the depression ulnar to the extensor digitorum tendon. |
| LI-5  (Yangxi) | Large Intestine | On the radial aspect of the wrist, with the thumb abducted, in a depression between the extensor pollicis longus tendon and brevis muscles (‘anatomical snuffbox’). |
| SP-3  (Taibai) | Spleen | On the medial aspect of the foot, in the depression proximal to the head of the 1st metatarsal bone, at the border of the red and white skin. |
| LR-3  (Taichong) | Liver | On the dorsum of the foot, between the 1st and 2nd metatarsal bones, in the depression proximal to the metatarsophalangeal joints and the proximal angle between the two bones. |
| KI-4  (Dazhong) | Kidney | On the medial aspect of the foot, anterior to the medial border of the Achilles tendon, superior to its insertion at the calcaneus. |
| BL-65  (Shugu) | Bladder | On the lateral aspect of the foot, in the depression proximal to the head of the 5th metatarsal bone. |
| GB-40  (Qiuxu) | Gallbladder | On the dorsum of the foot, in the depression anterior and inferior to the lateral malleolus, lateral to the extensor digitorum longus tendons. |
| ST-42  (Chongyang) | Stomach | On the dorsum of the foot, the point is bordered proximally by the 2nd and 3rd metatarsal bones and distally by the 2nd and 3rd cuneiform bones. |
